# Autoantibodies in the criteria of autoimmune diseases: is it sufficient to know that the test is positive?

**DOI:** 10.1016/j.jtauto.2022.100144

**Published:** 2022-01-18

**Authors:** Jan Damoiseaux

**Affiliations:** Central Diagnostic Laboratory, Maastricht University Medical Center, Maastricht, the Netherlands

For many autoimmune diseases criteria have been defined for diagnostic and/or classification purposes. Since autoantibodies are well established markers for autoimmune diseases, it is not surprising that the presence of disease-specific autoantibodies are incorporated in disease criteria. However, in such sets of criteria it is largely underestimated that immuno-assays for autoantibodies are very diverse and, importantly, not standardized. Although some assays enable reporting of results in international units because the assay has been calibrated on an internationally accepted standard preparation, the level of standardization achieved is very limited [1]. In short, this is due to the fact that the composition of the measurand, i.e., the autoantibody, is a heterogeneous mixture that differs between individual patients. In addition, disease criteria often simplify the result of an autoantibody assay as being either negative or positive. Since it is well recognized that higher levels of autoantibodies have increased clinical relevance [2], some disease criteria, for instance for celiac disease and rheumatoid arthritis [3,4], differentiate between low and high positive results. The distinction of the dichotomous outcome, as well as the distinction between low and high positive, is based on the cut-off value defined by the manufacturer of the assay. Diagnostic companies, however, utilize distinct strategies for defining the cut-off value [5]. Therefore, the choice of immuno-assay to be used in a laboratory will impact on the application of the disease criteria. This has been elegantly illustrated for rheumatoid factor in the classification criteria of rheumatoid arthritis [6].

The problem of standardization will be difficult to overcome, but the issue of differentially defining cut-off values could be addressed by harmonization of immuno-assays for autoantibodies [1]. Harmonization even goes beyond the definition of the cut-off and can be achieved at different levels, as best exemplified by the detection of anti-neutrophil cytoplasmic antibodies (ANCA) in ANCA-associated vasculitis [7]. First, the clinical manifestations that warrant ANCA-testing were clearly defined and enable an effective gating strategy. Second, there is international consensus that antigen-specific assays for both MPO- and PR3-ANCA are to be used as first-line screening assay. The possible subsequent testing-algorithm further defines the use of second-line confirmation or screening assays. Third, reporting of results has been extensively addressed. While also for ANCA cut-off settings largely differ between assays, the receiver operating characteristics curves for the assays used in a multicenter study were remarkably similar [8]. Consequently, the use of cut-off values defined by the level of specificity for all assays resulted in alignment of the test characteristics. Obviously, due to the lack of standardization, quantitative results remain different between assays. However, this can be overcome by expressing the results in likelihood ratios for test-result intervals, or even for individual test-results [9,10]. Finally, in a diagnostic setting, likelihood ratios of test-results facilitate interpretation of the test results in light of the clinical manifestations of the patient based on Bayes’ theorem. The pre-test probability, as defined by the clinical manifestations of the respective patient, in combination with the likelihood ratio of the test-result, enable to calculate the post-test probability, ideally in an automated algorithm, and this supports the clinician in the diagnostic work-up of the respective patient. Importantly, this approach is strongly embraced by distinct organizations involved in harmonization of autoantibody assays as well as the diagnostic industry [11].

In the current special issue of the Journal of Translational Autoimmunity, entitled “Autoantibodies in the disease criteria for systemic autoimmune diseases”, the positioning of autoantibodies in the disease criteria for distinct systemic autoimmune rheumatic diseases (SARD) will be addressed. Using the network of laboratory specialists and clinical immunologists/rheumatologists of the European Autoimmunity Standardisation Initiative (EASI) [12], authors were invited for contributions with selected topics to review the history of the positioning of autoantibodies in the disease criteria, the scientific evidence for including these autoantibodies, and to suggest possible adjustments in future versions of the criteria. Since anti-nuclear antibodies (ANA) not only have a central position in the criteria for most of the SARD, but are also relevant for autoimmune liver diseases [13], these diseases will be included as well. In particular the assays for ANA are prone to harmonization with respect to, for instance, used terminology and reporting of results. The International Consensus on ANA Patterns (ICAP) has made important achievements in terms of pattern definition and clinical relevance for the HEp-2 indirect immunofluorescent assays (IFA) [[14], [15], [16]]. Although the ANA result as such may already be part of the disease criteria, it is also used for reflex testing in order to define the antigen-specificity. Again, the use of such testing algorithms and of the applied multiplex immuno-assays are prone to harmonization and will be addressed in the contributions to this special issue. In the end, it is important that the lack of standardization and the options for harmonization for autoantibody assays are better recognized in the disease criteria of autoimmune diseases. This can be achieved by close collaboration between clinicians and laboratory specialists, one of the main goals of EASI, in the early stages of defining novel disease criteria, either for diagnosis or classification.

## Declaration of interests

The author declares that he has no known competing financial interests or personal relationships that could have appeared to influence the work reported in this paper.
